# Modulating proximal cell signaling by targeting Btk ameliorates humoral autoimmunity and end-organ disease in murine lupus

**DOI:** 10.1186/ar4086

**Published:** 2012-11-08

**Authors:** Jack Hutcheson, Kamala Vanarsa, Anna Bashmakov, Simer Grewal, Deena Sajitharan, Betty Y Chang, Joseph J Buggy, Xin J Zhou, Yong Du, Anne B Satterthwaite, Chandra Mohan

**Affiliations:** 1 University of Texas Southwestern Medical Center, Department of Internal Medicine, Division of Rheumatic Diseases, 5323 Harry Hines Blvd., Dallas, TX 75390-8884, USA; 2 Pharmacyclics, Inc., 995 East Arques Avenue, Sunnyvale, California 94085, USA; 3 University of Texas Southwestern Medical Center, Department of Pathology, 5323 Harry Hines Blvd., Dallas, TX 75390-8884, USA

## Abstract

**Introduction:**

Systemic lupus erythematosus is a chronic autoimmune disease characterized by an abundance of autoantibodies against nuclear antigens. Bruton's tyrosine kinase (Btk) is a proximal transducer of the BCR signal that allows for B-cell activation and differentiation. Recently, selective inhibition of Btk by PCI-32765 has shown promise in limiting activity of multiple cells types in various models of cancer and autoimmunity. The aim of this study was to determine the effect of Btk inhibition by PCI-32765 on the development of lupus in lupus-prone B6.Sle1 and B6.Sle1.Sle3 mice.

**Methods:**

B6.Sle1 or B6.Sle1.Sle3 mice received drinking water containing either the Btk inhibitor PCI-32765 or vehicle for 56 days. Following treatment, mice were examined for clinical and pathological characteristics of lupus. The effect of PCI-32765 on specific cell types was also investigated.

**Results:**

In this study, we report that Btk inhibition dampens humoral autoimmunity in B6.Sle1 monocongenic mice. Moreover, in B6.Sle1.Sle3 bicongenic mice that are prone to severe lupus, Btk inhibition also dampens humoral and cellular autoimmunity, as well as lupus nephritis.

**Conclusions:**

These findings suggest that partial crippling of cell signaling in B cells and antigen presenting cells (APCs) may be a viable alternative to total depletion of these cells as a therapeutic modality for lupus.

## Introduction

Systemic lupus erythematosus (SLE) is a systemic autoimmune disease characterized by the presence of autoantibodies, particularly against nuclear self-antigens. The recognition of these antigens by their cognate antibodies and the resultant deposition of immune complexes leads to the development of a chronic inflammatory state, which can have devastating effects on multiple end organ targets including the kidneys, the cardiovascular system, the skin, and the central nervous system. While the exact etiology of SLE is unclear, it is well established that SLE is a polygenic disorder with multiple dysregulated hematopoietic cell types contributing to the full-fledged disease state. To this end, it has been challenging to devise effective therapies for SLE given that multiple cellular and molecular checkpoints go awry in lupus. Bruton's tyrosine kinase (Btk) is intriguing as a potential therapeutic target in SLE given its proximal location in the B cell receptor (BCR) signaling cascade, as well as its previously described role in multiple myeloid cell types [1-5].

Many studies have focused on the role of B cells in lupus, and B cells have historically been a main target for SLE therapeutic interventions. Despite numerous studies and approaches to this problem, the goal of limiting the B cell response in SLE remains elusive [6]. Near-total elimination of B cells is problematic because it is becoming increasingly evident that B cells serve a number of other functions besides antibody (and autoantibody) production. These critical processes include T cell survival and anergy, promotion of regulatory T cells, and synthesis of anti-inflammatory cytokines, amongst others. Therefore, a more nuanced approach focused on dampening the B cell response may prove to be more beneficial in SLE.

Since B cell activation is achieved through BCR signaling, members of the BCR signaling cascade are of particular interest for study with regards to SLE. Btk has been a prime target due to its proximal location in the pathway and its direct link to B cell survival through NF-κB [7,8]. In humans, Btk plays a critical role in the development of B cells and subsequent antibody production, and mutation of the Btk gene results in X-linked agammaglobulinemia, which is characterized by low peripheral B cell numbers as well as low serum immunoglobulin titers [9]. Similarly, mutation or deletion of the Btk gene in mice leads to X-linked immunodeficiency (xid), characterized by a significant decrease in B1 and B2 B cells as well as significantly decreased serum immunoglobulin levels [9]. Although Btk is expressed in other hematopoietic lineages (but not T cells), the clinical phenotype of these genetic conditions is dominated by B cell immunodeficiency. It has long been appreciated that Btk is necessary for the production of autoantibodies in multiple murine models of lupus [10-12], and more recently it has been shown that constitutive activation of Btk in B cells results in the accumulation of autoreactive plasma cells [13]. Cell type-specific overexpression of Btk in B cells has recently been shown to lead to spontaneous germinal center and plasma cell formation, followed by autoantibody production [14]. However, even partial restoration of Btk by a low dose transgene in *lyn*/*btk*-double deficient mice results in abrogation of anti-nuclear antibodies [15]. Given the connection between B cell over-activity and various disease states, Btk has been investigated as a pathogenic target in multiple disease models. To this end, xid mice have been reported to be resistant to collagen-induced arthritis [16], and inhibition of Btk has been shown to be effective in treating various cancers as well as autoimmune diseases [17-20].

The NZM2410 mouse strain develops spontaneous lupus nephritis that shares a number of characteristics with human SLE [21]. Linkage analysis has uncovered several chromosomal intervals responsible for conferring lupus susceptibility in this strain [22]. By introgressing these intervals onto the healthy lupus-resistant C57BL/6 (B6) mouse strain, researchers have been able to determine the role of each locus in disease development. The presence of the *Sle1 *locus on the B6 background has been shown to be responsible for breaching immune tolerance to nuclear antigens. These mice display increased autoantibodies specific for chromatin, autoreactive T cells responding to histone epitopes, and enhanced expression of activation markers on T and B cells leading to functional defects in both cell types [23,24]. Meanwhile, the *Sle3 *locus introgressed onto the B6 background demonstrates dysregulated T cells as evidenced by hyperactivity and skewed CD4:CD8 ratios as a result of hyper-stimulatory antigen presenting cells (APCs) [25,26]. The *Sle3 *locus also confers some resistance to bacterial infections, further suggesting a role for this locus in the proper functioning of APCs [27]. Taken together, these data suggest a predominantly B cell-mediated role for the *Sle1 *locus and a predominantly innate immunity-targeted role for the *Sle3 *locus in lupus development. While the introduction of any single NZM2410-derived locus has not been shown to recapitulate complete disease, the presence of both the *Sle1 *locus and the *Sle3 *locus on the B6 background results in severe glomerulonephritis, reinforcing the notion that multiple genetic alterations in several cell types may be necessary to drive lupus pathogenesis [28].

Here, we have utilized mouse models bearing these lupus susceptibility loci to investigate the therapeutic potential of Btk in a spontaneous murine model of lupus nephritis by treating the mice with a small molecule Btk-inhibitor, PCI-32765; this is an irreversible inhibitor that binds covalently to Cys-481 of Btk with an IC50 of about 0.5 nM in biochemical assays. PCI-32765 is currently in phase II trials for treatment of B cell malignancies, and preliminary results demonstrate that approximately 60 to 70% of patients with relapsed and refractory chronic lymphocytic leukemia (CLL) or mantle cell lymphoma (MCL) respond to the drug [29,30].

## Materials and methods

### Mice

For these studies all mice were bred and housed at the University of Texas Southwestern Medical Center in Dallas, TX, USA. The studies were designed in conjunction with Pharmacylics, Inc. (Sunnyvale, CA, USA), and were conducted with the prior approval of the University of Texas Southwestern Medical Center Institutional Animal Care and Use Committee. Based on the typical age of phenotype development in these models 8-month-old male and female B6.*Sle1 *and pre-disease 4-month-old female B6.*Sle1*.*Sle3 *mice were used for the experiments. Autoantibody levels were examined prior to the start of treatment to ensure the mice were at similar stages of disease. Both sets of mice were treated for two months and then sacrificed to ensure that mice did not succumb to disease before the data could be collected.

### Drug formulation

PCI-32765 (Pharmacyclics Inc.) was formulated in sterile-filtered 1% HP-beta-cyclodextrin in distilled water. The drug was provided in drinking water at 0.16 mg/mL, which is an estimated dose of 30 mg/kg calculated based on average water consumption. The vehicle was sterile-filtered 1% HP-beta-cyclodextrin in distilled water. The amount of drug- or vehicle-containing water consumed was approximately 3.5 to 4.0 mL per mouse per day and was similar between groups and cages (data not shown). Splenocytes were isolated from B6.Sle1.Sle3 mice treated with PCI-32765 after sacrifice on day 56 and the pharmaco-dynamic probe assay for Btk was performed as previously described [17].

### Histopathology

Kidneys isolated from 6-month-old B6.Sle1.Sle3 mice were cut laterally and fixed in phosphate-buffered 10% formalin, processed in paraffin blocks and cut into 5-μm sections. Sections were stained with periodic-acid Schiff (PAS) or H&E and examined by light microscopy for evidence of inflammation or tissue damage. At least 100 glomeruli per section were examined and scoring (0 to 4) of renal damage was done by a pathologist blinded to the study, as detailed previously [31].

### Autoantibody ELISA

Ninety-six-well plates were pre-coated with methylated BSA before the addition of double stranded DNA, single stranded DNA, histones, or double stranded DNA and then histones (nucleosomes) as detailed previously [31]. After incubation, the plates were washed, sera from the study mice were added to the wells, and the plates were again incubated. After washing off the unbound sera, alkaline phosphatase-conjugated goat anti-mouse IgG or IgM antibodies were added. Autoantibody titers were determined by the absorbance read at 405 nm on an ELx808 plate reader (BioTek, Winooski, VT, USA).

### Flow cytometry

Spleens and kidneys were isolated from mice following sacrifice and perfusion. For the spleens, a single cell suspension was obtained by crushing the spleen through a 100-micron mesh filter and washing the cells. Red blood cells were lysed using PharmLyse (BD Biosciences, San Diego, CA, USA). Kidneys were minced with a razor blade and treated with collagenase for 30 minutes at 37°C with shaking. Following collagenase treatment, cells were immediately placed on ice and the collagenase activity was stopped by dilution with media containing fetal bovine serum. Red blood cells were lysed in a similar manner to splenocytes and cell clumps were disrupted by syringe and then forced through a 100-micron filter. Cells were stained with antibodies against AA4.1, B220, CD4, CD5, CD8, CD11b, CD11c, CD21/35, CD23, CD34, CD45, CD62L, CD69, CD138, c-Kit, GL-7, Ly6C, Ly6G (BD Biosciences) and F4/80 (Invitrogen, San Diego, CA). All samples were run on an LSRII flow cytometer (BD Biosciences) in the University of Texas Southwestern Medical Center Flow Cytometry Core.

### Statistics

All statistical analyses were done by two-tailed Student's *t*-test with Welch's correction.

## Results

### PCI-32765 checks the increase in autoantibodies inherent to the Sle1 locus

**Figure 1:**
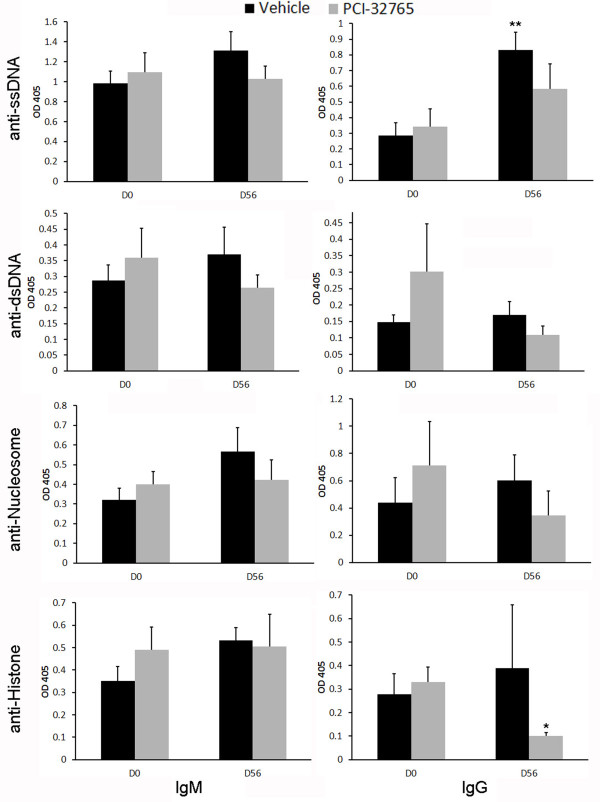
**PCI-32765 dampens the production of anti-nuclear autoantibodies in B6.Sle1 mice**. The indicated autoantibody ELISAs were performed on sera isolated at the indicated day of treatment from mice either treated with PCI-32765 (black bars, *n *= 5) or a vehicle (grey bars, *n *= 4). All values are the optical density (OD) readings at 405 nm wavelength. *P < 0.05, **P < 0.01 compared to day-0 values measured in the same mice. D0, day zero; D56, day 56.

While C57BL/6 mice do not spontaneously develop lupus, the presence of the *Sle1 *locus from the NZM2410 lupus-prone strain results in the development of autoantibodies, especially anti-histone and anti-nucleosome antibodies, as well as hyperactivated T and B cells [23,24]. Given that the *Sle1 *locus has previously been shown to be related to a defect in B cell tolerance, we targeted the proximal BCR-mediated signaling molecule Btk by treating B6.*Sle1 *mice with PCI-32765, to examine the impact of disrupting BCR-signaling on autoantibody development. Although IgM isotype anti-ssDNA, anti-nucleosome, and anti-histone antibodies did not show significant differences following treatment, PCI-32765-treated mice demonstrated reduced levels of IgG isotype anti-histone, anti-nucleosome and anti-dsDNA levels, with the latter attaining statistical significance (69% reduction, *P *< 0.05), compared to day 0 levels in the same mice (Figure 1). In contrast, circulating levels of all measured autoantibodies increased in vehicle-treated mice over the same time period (Figure 1). Taken together these data indicate that Btk plays an important role in both autoantibody production and isotype switching, with a more profound impact on class-switched IgG autoantibodies. These data are similar to previous genetic studies demonstrating that reduced Btk gene dosage preferentially affects IgG antibodies in 56R anti-DNA transgenic mice but both IgM and IgG antibodies in *lyn*-/- mice [15,32]. Interestingly, the anti-histone and anti-nucleosome titers that are most affected by PCI-32765 treatment resemble the anti-nucleosome seroprofile previously attributed to *Sle1 *[23,24].

### Inhibition of Btk results in a less severe Sle1 phenotype

**Figure 2:**
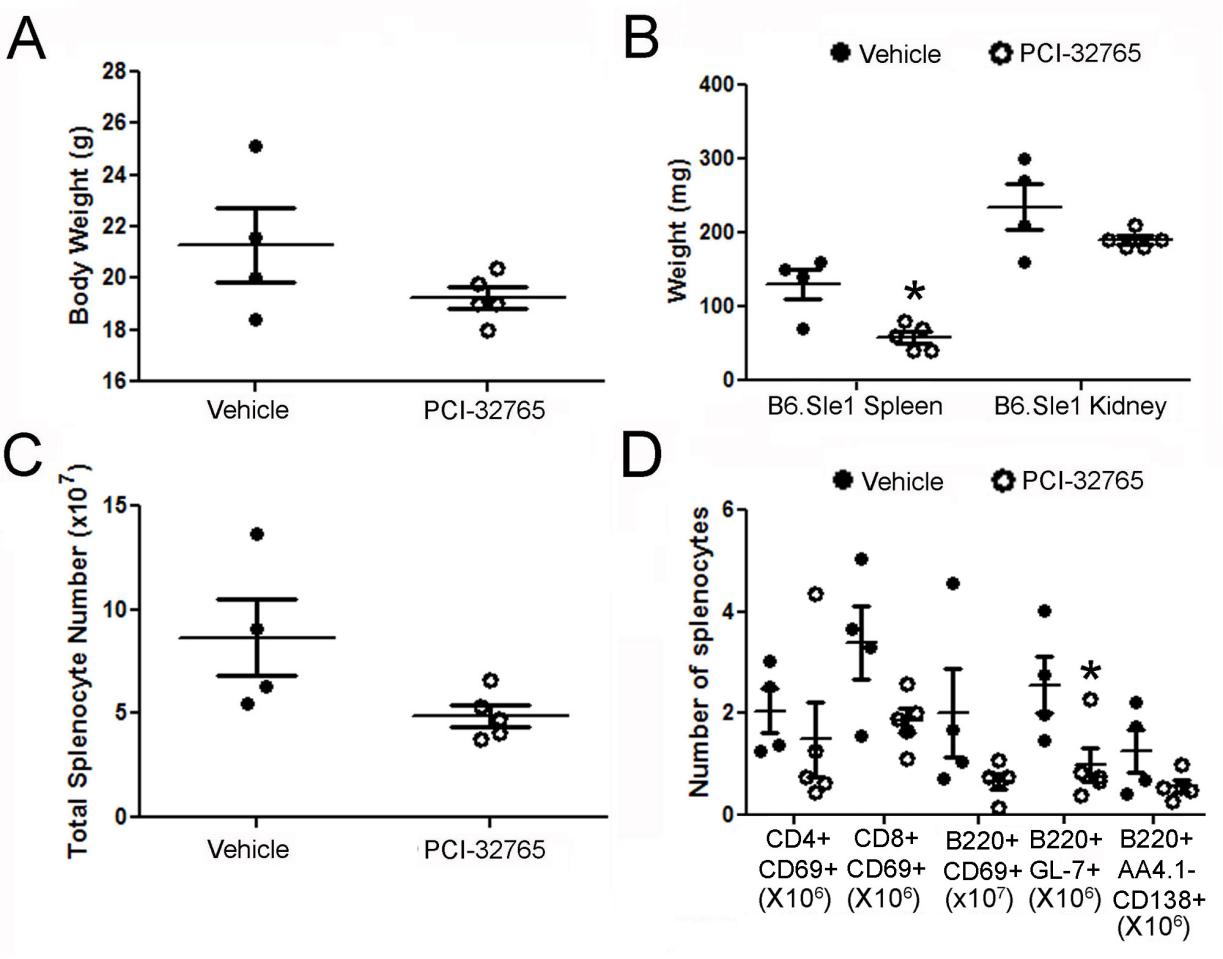
**Treatment with PCI-32765 affects spleen size and lymphocyte activation in B6.Sle1 mice**. (**A**) Mice that received PCI-32765 are of similar weight compared to vehicle treated mice. All mice were weighed before sacrifice. (**B-C**) PCI-32765 reduces splenomegaly, but not kidney size, as measured by weight or cell number. Spleens and kidneys isolated from mice treated with PCI-32765 (*n *= 9) or vehicle (*n *= 8) for 56 days were weighed prior to the preparation of a single cell suspension of each tissue. Cells were then counted on a Cellometer Auto M10 automated cell counter (Nexcelom Bioscience, Lawrence, MA, USA). (**D**) Inhibition of Btk results in decreased lymphoid activation. Single cell suspensions were prepared from isolated spleens and stained for flow cytometry analysis of the indicated lymphocyte populations and activation status (CD69+). Each data point represents one mouse. Line represents mean ± standard error of the mean. *P < 0.05, **P < 0.005. Data were compared by two-tailed Student's *t*-test with Welch's correction.

Although the *Sle1*-bearing mice do not develop full blown lupus-like disease, the *Sle1 *locus has been shown to elicit lymphocyte activation and splenomegaly [23]. B6.*Sle1 *mice treated with PCI-32765 have no significant change in total body weight compared to vehicle-treated mice (Figure 2A); however the PCI-32765-treated mice display a 43.3% decrease in spleen size and total splenocytes compared to the vehicle-treated mice (4.90 × 10^7^vs. 8.65 × 10^7 ^splenocytes, Figure 2B-C). This reduction in cell number coincides with a decrease in activated CD4+ (28%) and CD8+ (46%) T-cells, activated B-cells (53%), germinal center B-cells (62%, *P *= 0.05, B220+GL7+), and plasmablasts (56%) relative to the vehicle group (Figure 2D). However, the number of myeloid cells, including dendritic cells (CD11b+CD11c+), macrophages (CD11b+F4/80+), and neutrophils (CD11b+Ly6G+) is not significantly changed (data not shown). Taken together, these data suggest that Btk plays an important role in regulating immune cell activation in the context of lupus, affecting multiple cell lineages.

### PCI-32765 delays production of circulating autoantibodies in Sle1.Sle3 bicongenic mice

**Figure 3:**
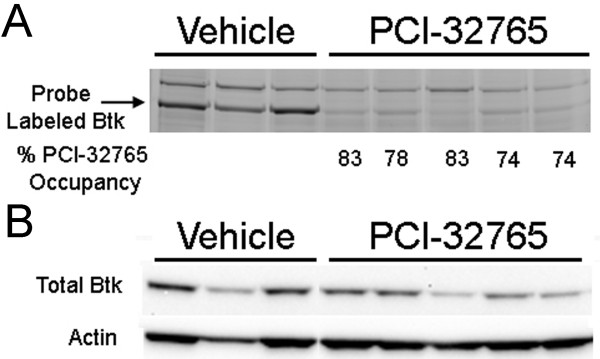
**Inhibition of Bruton's tyrosine kinase (Btk) by PCI-32765**. (**A**) Fluorescent gel scan of splenic lysates from mice that had been treated with PCI-32765 or vehicle. Cells were incubated with the affinity probe PCI-33380, a fluorescently tagged derivative of PCI-32765, prior to lysis and visualization by SDS-PAGE. Binding of PCI-33380 indicates unbound Btk was available in the cells. The arrow indicates the predominant band labeled by the probe (at approximately 76 kDa, the expected molecular weight of Btk). The percentage of occupancy is based on densitometry relative to the vehicle-treated samples. (**B**) Total Btk expression was determined by western blot.

Though *Sle1 *by itself is not sufficient for full-blown lupus to ensue, bicongenics bearing both *Sle1 *and *Sle3 *develop lupus nephritis, likely as a result of a cumulative impact on multiple cell types including both B cells and APCs [33]. Given that the B6.*Sle1*.*Sle3 *mouse strain develops more severe disease with spontaneous glomerulonephritis around 6 months of age, we next treated 4-month-old (pre-disease) B6.Sle1.Sle3 mice for 2 months with either PCI-32765 (*n *= 9) or a vehicle (*n *= 8) to determine if disease progression could be delayed or halted. Following treatment, splenocytes from the mice were isolated and assayed to ensure that PCI-32765 was binding Btk as previously reported (Figure 3A) [17]. Mice that had been treated with the vehicle displayed a thick band representative of unbound Btk. However, B6.Sle1.Sle3 mice that received drinking water containing PCI-32765 had significantly lighter bands, suggesting that Btk had been bound by PCI-32765 in these mice. We determined by densitometry that an average of 78.4% of the Btk was bound by PCI-32765 in the treatment group compared to the vehicle-treated mice (Figure 3A). The total expression of Btk was not affected in the PCI-32765-treated mice (Figure 3B).

**Figure 4:**
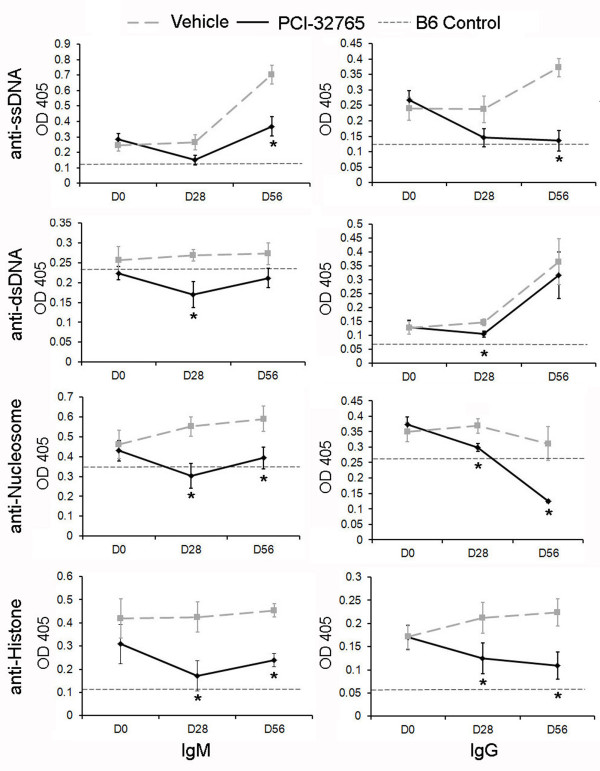
**Inhibition of Btk limits increase of IgM isotype autoantibodies and results in decrease of IgG isotype autoantibodies in B6.Sle1.Sle3 mice**. The indicated autoantibody ELISAs were performed on sera routinely isolated from mice either treated with PCI-32765 or vehicle. All values are the optical density (OD) readings at 405 nm wavelength. Thick grey dashed line indicates vehicle, black line indicates PCI-32765. Thin dashed line indicates serum antibodies in 2-month-old lupus-resistant B6 sample indicative of negative control. All data points represent the mean ± standard error of the mean. *P < 0.05. Samples were compared by two-tailed Student's *t*-test with Welch's correction. D0, day zero, D28, day 28; D56, day 56.

As noted in the B6.Sle1 treatment studies, Btk blockade also dampened autoantibody production in the bicongenic lupus strain. By day 28, the mice receiving the Btk-inhibitor displayed significantly reduced levels of both IgM and IgG anti-nucleosome and anti-histone antibodies (Figure 4). This reduction of circulating anti-nucleosome and anti-histone antibodies was sustained through day 56, by which time point anti-ssDNA (but not anti-dsDNA) antibodies were also reduced (Figure 4).

### PCI-32765 reduces splenomegaly and leukocyte activation in lupus-prone Sle1.Sle3 mice

**Figure 5:**
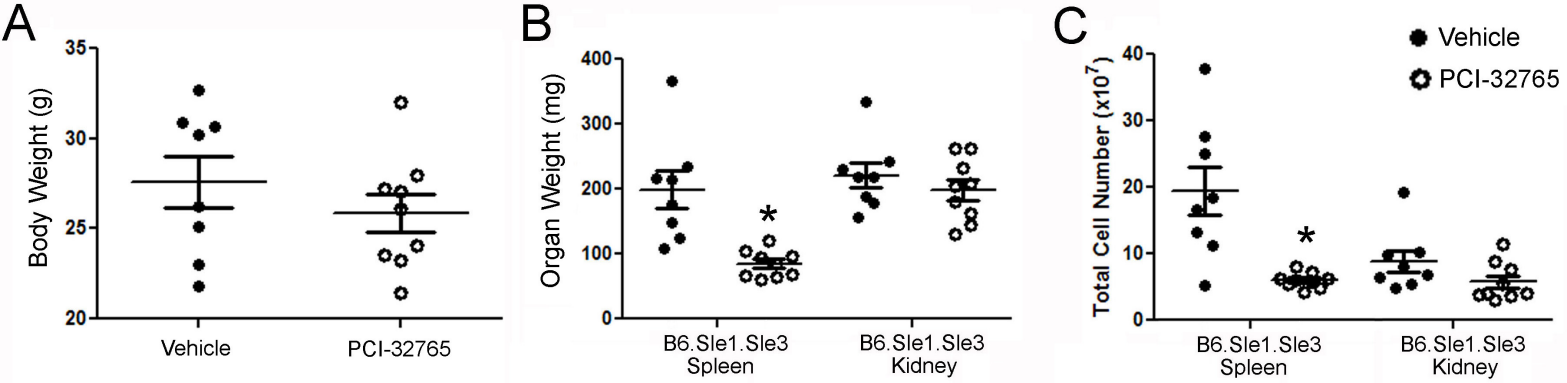
**Treatment of B6.Sle1.Sle3 mice with PCI-32765 results in reduction of splenomegaly**. (**A**) PCI-32765 has no effect on mouse weight. All mice were weighed before sacrifice. (**B-C**) PCI-32765 reduces splenomegaly, but not kidney size, as measured by weight or cell number. Spleens and kidneys isolated from mice treated with PCI-32765 (*n *= 9) or vehicle (*n *= 8) were weighed prior to the creation of a single cell suspension of each tissue. Cells were then counted on a Cellometer Auto M10 automated cell counter (Nexcelom Bioscience). Each data point represents one mouse. Line represents mean ± standard error of the mean. *P < 0.05, **P < 0.005. Data were compared by two-tailed Student's *t*-test with Welch's correction.

After two months of treatment, spleen weights of the sacrificed animals were reduced by 58% in the treated mice (84.4 mg vs. 199 mg, *P *= 0.005, Figure 5B). Splenic cell count was reduced by 70% (1.94 × 10^8^vs. 5.9 × 10^7^, *P *= 0.008) in these mice compared to the vehicle-treated mice (Figure 5C). In contrast, the size and cellularity of the kidneys were not significantly reduced by PCI-32765 treatment, nor was the overall body weight (Figure 5A-C).

Flow cytometric analysis revealed significant decreases in splenic populations of multiple cell types in mice treated with PCI-32765 (Table 1), including total splenic B cells (71.5%, *P *< 0.05), activated B cells (74.9%, *P *< 0.01), and B cells from multiple stages of development including T1 (64%, *P *< 0.05) and T2 transitional B cells (78.6%, *P *< 0.01), mature marginal zone (74.6%, *P *< 0.05) and follicular B cells (63.2%, *P *< 0.05), and plasmablasts (74.5%, *P *< 0.01). Given that Btk has also been described in multiple myeloid cell types, these cell populations were also examined in mice treated with PCI-32765 (*n *= 9) compared to vehicle-treated mice (*n *= 8). Significant decreases were observed in myeloid dendritic cells (79.2%, *P *< 0.005), macrophages (76.4%, *P *< 0.01), neutrophils (68.1%, *P *< 0.01), mast cells (80.3%, *P *< 0.02), and CD11b+Ly6C+CD62L- (73.6%, *P *< 0.05) cells, which may represent newly infiltrating inflammatory monocytes, as summarized in Table 1. Interestingly, despite the lack of a T cell specific role for Btk, multiple splenic T cell populations from both T helper and cytotoxic T cell lineages were also impacted by treatment with PCI-32765. These significantly reduced T cell populations included total T helper cells (82.7%, *P *< 0.01), activated T helpers (83.6%, *P *< 0.005), and both central (84.6%, *P *< 0.05) and effector (80.5%, *P *< 0.01) memory T helper cells as well as total (80.8%, *P *< 0.01), central memory (75.5, *P *< 0.01) and effector memory (80.1%, *P *< 0.01) cytotoxic T cells (Table 1). Strikingly, despite the significant decrease in splenic size following PCI-32765 treatment, there was no difference in the number of naïve CD4+ T helper or naïve CD8+ cytotoxic T cell populations. Taken together, these data suggest that treatment with PCI-32765 skews T cells towards a less activated phenotype, likely as a result of decreased B cell and APC activation.

**Table 1:** Analysis of surface phenotype of splenocytes

	Cell Number/Spleen				
**B cells**	**Average** **Vehicle**	**SEM Vehicle**	**Average PCI-32765**	**SEM PCI-32765**	***P*-value**

B220+	3.97E+07	1.13E+07	9.06E+06	2.76E+06	*
Activated, B220+CD69+	3.71E+06	9.33E+05	5.29E+05	9.19E+04	**
T1, B220+AA4.1+CD23-	1.64E+07	5.90E+06	2.95E+06	8.72E+05	*
T2, B220+AA4.1+CD23+	1.60E+07	3.42E+06	4.02E+06	1.79E+06	**
Marginal zone, B220+AA4.1-CD23-	1.99E+06	5.05E+05	5.25E+05	1.92E+05	*
Follicular, B220+AA4.1-CD23+	5.13E+06	1.89E+06	8.82E+05	2.18E+05	*
Plasmablast, B220+AA4.1-CD138+	3.62E+06	9.24E+05	4.36E+05	7.13E+04	**
Germinal center, B220+GL7+	8.61E+06	4.52E+06	8.72E+05	3.38E+05	NS
B1-like, B220+CD5+	1.01E+06	2.62E+05	2.60E+05	3.93E+04	*
**Myeloid**					
Myeloid DC, CD11b+CD11c+	1.33E+07	2.76E+06	2.19E+06	4.28E+05	***
Macrophage, CD11b+F4/80+	1.32E+07	3.11E+06	2.15E+06	3.88E+05	**
CD11b+Ly6C+	1.74E+06	5.54E+05	3.40E+05	6.72E+04	*
Neutrophils, CD11b+Ly6G+	1.50E+07	3.51E+06	2.41E+06	4.16E+05	**
Mast cell, CD34+c-Kit+	3.74E+06	9.87E+05	7.36E+05	2.54E+05	*
**T cells**					
CD4+	2.31E+07	4.00E+06	9.83E+06	9.76E+05	**
Activated, CD4+CD69+	8.29E+06	1.36E+06	2.64E+06	1.01E+06	***
Naïve, CD4+CD44-CD62L+	9.73E+05	2.34E+05	5.68E+05	9.45E+04	NS
Central memory, CD4+CD44+CD62L+	5.11E+06	7.89E+05	2.84E+06	3.49E+05	*
Effector memory, CD4+CD44+CD62L-	1.56E+07	3.04E+06	6.00E+06	7.58E+05	**
CD8+	2.01E+07	3.86E+06	5.29E+06	8.23E+05	**
Activated, CD8+CD69+	8.57E+06	1.60E+06	2.54E+06	1.17E+06	**
Naïve, CD8+CD44-CD62L+	6.18E+03	3.33E+03	3.34E+03	2.65E+03	NS
Central memory, CD8+CD44+CD62L+	5.64E+06	1.38E+06	9.08E+05	1.65E+05	**
Effector memory, CD8+CD44+CD62L-	1.45E+07	2.87E+06	4.38E+06	8.23E+05	**

Quantitative analysis of B6.Sle1.Sle3 splenic cell populations as determined by flow cytometry. All data are expressed as the mean (Average) and standard error of the mean (SEM) of the data for the PCI-32765-treated (*n *= 9) or vehicle-treated (*n *= 8) splenocytes, which were compared by two-tailed Student's *t*-test. *P ≤ 0.05, **P ≤ 0.01, ***P ≤ 0.005, compared to vehicle; NS, not significant.

### Reduced renal damage and lymphocyte infiltration in PCI-32765-treated mice

**Figure 6:**
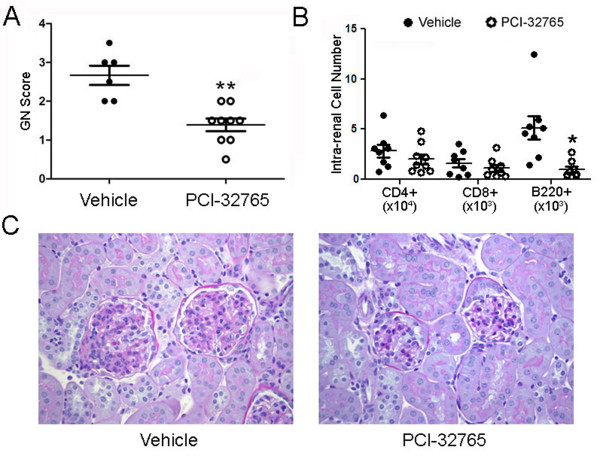
**Decreased renal destruction and lymphocyte infiltration in B6.Sle1.Sle3 mice after inhibition of Btk signaling**. (**A**) Inhibition of Btk by PCI-32765 reduced renal damage. Paraffin-embedded kidneys were sectioned and stained with H&E before being scored by a pathologist blinded to the sample identity. (**B**) Decreased number of renal lymphocytes in mice that received PCI-32765. Single cell suspensions were prepared from kidneys isolated from mice treated with PCI-32765 or vehicle. After staining with antibodies specific for the indicated cell populations, percentage cell numbers were multiplied by the total intra-renal cell number as determined by a Cellometer Auto M10 automated cell counter (Nexcelom Bioscience). Each data point represents one mouse. Line represents mean ± standard error of the mean. *P < 0.05, **P < 0.005. Data were compared by two-tailed Student's *t*-test with Welch's correction. (**C**) Histopathological signs of renal damage. Shown are representative photomicrographs of period acid Schiff (PAS)-stained kidney sections isolated from PCI-32765-treated (*n *= 9) or vehicle-treated (*n *= 6) mice. All images were taken at 200 × total magnification. GN, glomerular nephritis.

To determine if treatment with PCI-32765 was able to limit renal damage, paraffin-embedded kidneys from B6.Sle1.Sle3 mice treated with either PCI-32765 (*n *= 9) or vehicle (*n *= 6) were sectioned, stained and scored by a pathologist who remained blind to sample identities. While the kidneys from the vehicle-treated mice had moderate damage (glomerular nephritis (GN) score 2.67 ± 0.25, *n *= 6), the PCI-32765-treated mice displayed significantly less renal damage (GN score 1.39 ± 0.16, *n *= 9, *P *< 0.02, Figure 5A, C). There was also a corresponding decrease in the number of infiltrating lymphocytes in the kidneys. Despite similar numbers of total intra-renal cells in both groups (Figure 6A), there were significantly fewer B220+ B-cells (59% decrease, *P *< 0.01) in the kidneys of B6.Sle1.Sle3 mice that received PCI-32765 compared to mice that received the vehicle (Figure 6B).

## Discussion

When cells are initially confronted with antigens, each cell is at a crossroads from which the cell can either become activated and begin an immune response, or be eliminated through anergy induction or apoptosis. Autoimmunity at its most basic level, is characterized by the improper response to self-antigens leading to activation of an immune response. Historically, treatment for SLE has been limited to general immune suppressive drugs, including corticosteroids, anti-malarial drugs, and cyclophosphamide, all of which are limited in their efficacy and are associated with deleterious side effects [34]. The recent US Food and Drug Administration (FDA) approval of belimumab, an inhibitor of B lymphocyte stimulator (BLyS), for the treatment of SLE patients has highlighted a new wave of signaling targets that are being therapeutically exploited with some encouraging results in lupus. These novel candidates include a variety of intracellular proteins such as calmodulin kinase, calcineurin, heat shock protein 90 and Rho, as reviewed [34,35]. In B cells, one potential mechanism for accomplishing this is to disrupt BCR signaling, as the BCR is responsible for delivering maturation signals to immature and mature B cells. Inhibition of the BCR-component, spleen tyrosine kinase (Syk), has shown promise in attenuating skin and renal manifestations of lupus in certain mouse models [36], although translation to human trials may be complicated by the expression of Syk on a number of cell types. Another such target within the BCR-signaling cascade is Btk.

Btk is a particularly attractive target in this signaling cascade because it has a specific role in B cell activation and survival. While xid- and Btk-deficient mice display a marked reduction in mature B cells in the periphery, these mice have normal numbers of pre-B and immature B cells [37,38]. Furthermore, the loss of Btk function dampens the ability of B cells to respond to signals from cell surface receptors including the BCR, IL-10R, RP105, CD38, and toll-like receptors (TLRs), amongst others [39-43]. Thus, the addition of a Btk transgene to Btk-deficient mice restores the mature B cell population but still does not restore full response to BCR signals [44]. B cell response to T-independent antigens are also reliant on Btk in a dose-dependent fashion, as partial restoration of Btk restores normal B cell response to type-II viral antigens but not 2,4,6-trinitrophenol (TNP)-Ficoll. Taken together, these data suggest that modulation of Btk levels can differentially affect B-cell differentiation, survival, and function without eliminating the majority of mature B-cells. Therefore, our goal was to modulate, rather than completely block, BCR signaling. Such inhibition of BTK with PCI-32765 has recently been shown to be efficacious in patients with B cell malignancies [19,45] as well as in mice with autoimmune diseases [17,20].

Previously the *Sle1 *locus has been identified as being crucial in breaking peripheral B cell tolerance [46]. Thus, the B6.*Sle1 *mouse is a particularly attractive model for testing the ability of Btk inhibition to modulate the humoral autoimmunity associated with lupus. Upon treatment with PCI-32765, inhibition of Btk resulted in a dampening of the *Sle1*-associated phenotype at both the cellular and serological levels. It is clear from these studies in B6.*Sle1 *mice that Btk in B-cells is a critical B-cell activation node in lupus development. While B6.*Sle1 *mice provide a model to investigate the role of humoral immunity in SLE, single congenic *Sle1 *mice do not fully develop lupus nephritis. Hence, the B6.*Sle1*.*Sle3 *bicongenic mouse model, which develops a more robust lupus-like phenotype was next utilized. This model was well suited to this study given the previously reported roles of Btk in myeloid cell activation, and established myeloid cell population defects attributed to the *Sle3 *locus. Importantly, PCI-32765 treatment markedly reduces spleen size and renal infiltration of leukocytes as well as renal damage in B6.*Sle1*.*Sle3 *mice. In these studies, we have observed a decrease in all peripheral B cell populations in the PCI-32765 treated mice compared to the vehicle, although B-cell development appears to be relatively normal. Consistent with genetic data from Btk^low ^mice, these data suggest that a partial Btk signal is enough to permit B-cell development while still managing to regulate the overall number of mature and activated B cells [15]. Btk inhibition led to significantly reduced numbers of splenic plasmablasts (Table 1), consistent with previous findings that Btk is necessary for antibody production in a gene dosage-dependent manner [13-15,32,47].

Contrary to a previous report, which suggested that Btk repressed dendritic cell maturation and increased T helper cell activation by dendritic cells [48], we found that PCI-32765 treatment decreases splenic myeloid dendritic cells as well as T cell activation (Table 1); these data may suggest a dose-sensitive context for Btk in dendritic cells, similar to previous experiments with Btk^low ^mice in B cells [12,15,44]. We have also demonstrated that treatment with PCI-32765 significantly decreases the number of splenic mast cells, corresponding with previous data that demonstrate a role for Btk in mast cell activation [49]. Taken together, these decreases in multiple immuno-competent cell populations, as well as the dampening of lymphocyte activation, underscore the ability of PCI-32765 to curtail the aberrant cellular activation that drives lupus pathogenesis.

Of note, inhibition of Btk also results in a decrease in B-cell infiltration into the kidney. It has previously been reported that intra-renal B-cells contribute to kidney damage and interstitial inflammation [50-52]. Likewise, renal damage was reduced in B6.Sle1.Sle3 mice that received PCI-32765 compared to the vehicle, as evidenced by decreased GN score and decreased glomerular size (Figure 6A, C). However, given that splenic myeloid cell populations are also decreased (Table 1) it is possible that some of the beneficial effects of Btk inhibition may arise from its impact on the myeloid cell compartment, which also entails Btk signaling for optimal functioning. It has previously been shown that Btk is involved in various myeloid cell-mediated processes in mast cells [1], neutrophils [2,3], and macrophages [4,5], including bacterial clearance, production of reactive oxygen species (ROS), and TLR signaling. Moreover, PCI-32765 has previously been shown to exert effects on myeloid cells in the prevention of experimental arthritis [20].

In summary, we have shown that treatment of lupus-prone mice with PCI-32765 results in greater than 75% inhibition of Btk, which is sufficient to reduce autoantibody titers, similar to Btk^low ^mice, which express 25% of endogenous Btk levels [12,53]. This is accompanied by marked reduction in both the number and the activation status of T and B cells as well as the number of myeloid cells. Since T cells lack Btk expression, it seems likely that the decreased number and activation status of T cells is a result of decreased activation cues from B cells and APCs. Meanwhile, it seems plausible that the decrease in B cell and APC numbers may be caused by either decreased expansion resulting from suboptimal activation, increased apoptosis, or both. Previous reports lend support to both mechanisms as Btk has been shown to be critically important in both myeloid [1-5] and B cell activation [13,14,50] as well as in the negative regulation of apoptotic processes. Relevant to our data, Btk is required for BCR-mediated NF-κB pro-survival signaling [7,8,54], and is an upstream regulator of the anti-apoptotic protein bcl-xL in B cells following IgM stimulation [55]. Btk is also necessary for the survival of macrophages following inflammatory stimulation [56], and is a regulator of the ROS response in both B cells [57] and neutrophils [58]. Furthermore, *in vitro *PCI-32765 treatment of CD19+ cells isolated from patients with CLL resulted in increased apoptosis in a dose-dependent manner [19]. Thus, subdued proliferative expansion as well as enhanced apoptosis could both have contributed to the cellular changes observed following Btk inhibition in lupus, and this warrants further systematic evaluation.

Taken together these data suggest that Btk signaling, as a key mediator of B cell activation, antibody production, and myeloid cell activation, is vital to the pathogenesis of SLE. Modulating BCR-mediated and APC signaling and/or survival by Btk inhibition results in decreased lymphoid activation and expansion, without total depletion. Given that total B cell depletion may not be optimal in SLE [6], fine-tuning the responsiveness of these cells as opposed to eliminating them completely may be a more viable therapeutic strategy in SLE. Therapeutically reducing the number of B cells to blunt autoimmunity comes with the inherent risk of eliminating the natural ability to produce antibodies against invading pathogens. Given that B cell and plasmablast populations are decreased but not completely eliminated following PCI-32765 treatment, it is likely that some degree of humoral immunity is preserved in these animals; nevertheless, further investigation is necessary to determine the full impact of PCI-32765 treatment on both the primary and secondary immune responses.

## Conclusions

PCI-32765 treatment significantly decreased the amount of free Btk, and this dampened the development and activation of multiple peripheral cell types in the immune system, and decreased lupus severity as indicated by reduced autoantibody levels, splenomegaly, and renal disease. Therefore, targeting Btk to decrease downstream signaling in multiple cell types may be an effective therapeutic approach in SLE.

## Abbreviations

APC: antigen presenting cell; BCR: B cell receptor; BLys: B lymphocyte stimulator; BSA: bovine serum albumin; Btk: Bruton's tyrosine kinase; CLL: chronic lymphocytic leukemia; ELISA: enzyme-linked immunosorbent assay; GN: glomerular nephritis; H&E: hematoxylin and eosin; IL: interleukin; MCL: mantle cell lymphoma; PAS: period acid Schiff; ROS: reactive oxygen species; SLE: systemic lupus erythematosus; Syk: spleen tyrosine kinase; TLR: toll-like receptor; TNP: 2,4,6-trinitrophenol; xid: x-linked immunodeficiency.

## Competing interests

BYC and JJB are employed by Pharmacylics Inc. and hold stock and/or stock options related to Pharmacyclics Inc. This study was partially funded by Pharmacylics Inc., which holds patents and patent applications related to PCI-32765. All other authors have no competing interests to disclose.

## Authors' contributions

JH supervised collection of mouse samples, acquired flow cytometry data, analyzed flow cytometry and immunoassay data, and drafted the manuscript. KV assisted in collection of mouse samples and carried out the immunoassays. AB, SG, and DS monitored mouse health and compound supply and assisted in collection of mouse samples. BYC assisted in study design, data interpretation, and manuscript preparation. JJB and ABS helped with data interpretation and manuscript preparation. XJZ and YD acquired and analyzed histology data. CM participated in the conception of the study, supervised the study and drafted the manuscript. All authors have read and approved the manuscript for publication.
